# Supplementary material for: UK Women’s Views of the Concepts of Personalised Breast Cancer Risk Assessment and Risk-Stratified Breast Screening: A Qualitative Interview Study
Source: Cancers (Basel). 2021 Nov 19;13(22):5813. doi: 10.3390/cancers13225813 (PMC8616436; doi:10.3390/cancers13225813)
Supplement: Supplementary file 1 [file cancers-13-05813-s001.zip › Supplementalmaterial3.pdf]

Supplementary Material S3: Tabulated outline of the stages Framework Analysis for synthesising interview data to define main themes.

Organisational themes	*Codes*	*Main themes*	*Sub themes*
**Experience**	Health & lifestyle behaviours		
	Personal health status		
	BC attitudes & experience		
**Knowledge & understanding**	BC knowledge, perceived severity/consequences		
	Perceived risk, control and knowledge of RFs	**Risk perceptions and acceptability of personalised risk assessment**	• Perceived risk<br>• Understanding of PRA<br>• Willingness to receive risk feedback
	Understanding/misconceptions - PRA		
	Understanding of rationale for RSBS		
**Intentions**	Providing data for PRA (classic; PRS & MD)		
	Willingness to receive PR feedback		
	Attitudes towards extended age-range (HR)	**Ways of responding**	A typology:<br>• Overall acceptors<br>• More is better<br>• Screening sceptics
	Attitudes towards increased frequency (HR: 3 & 4 risk-groups)		
	Attitudes towards MR (4 risk-groups)		
	Attitudes towards AR (3 & 4 risk-groups)		
	Attitudes towards shorter age-range (LR)		
	Attitudes towards less frequent screening (LR: 3 & 4 risk-groups)		
	Responses to No screening (v.LR + 4 risk-groups)		
**Manageability**	Influence of lifestyle and chemoprevention on RSBS scenarios	**Influence of ladder of risk**	
	Influence of ladder of risk		
	Need for information & support	**Concerns and conditions of acceptability**	• Information support<br>• Breast awareness support<br>• Integration of PRA and RSBS
	Responses to screening harms & impact on RSBS scenarios		
**Choice & Responsibility**	Personal vs. shared health responsibility for risk-management		
	Attitudes towards lifestyle change		
**Trust & Confidence**	Communication with HPs and help-seeking		
	Attitudes towards medical research/innovation & health technology		
	Trust in accuracy of risk prediction models		
	Ethical & society implications of PRA & RSBS		
	NHS resources and trust in health providers		
**Overall impressions**	BPS versus RSBS	**Perceived effectiveness: RSBS vs. current NHSBS**	
	Screening modalities		
	Misunderstandings		
	Organisational issues & concerns		

Abbreviations: average risk (AR); breast cancer (BC); breast screening programme (BSP); health professional's (HPs); high-risk (HR); low-risk (LR); ,moderate risk (MR); personal risk assessment (PRA); risk-factors (RFs) and risk-stratified breast screening (RSBS).
