# Supplementary material for: UK Women’s Views of the Concepts of Personalised Breast Cancer Risk Assessment and Risk-Stratified Breast Screening: A Qualitative Interview Study
Source: Cancers (Basel). 2021 Nov 19;13(22):5813. doi: 10.3390/cancers13225813 (PMC8616436; doi:10.3390/cancers13225813)
Supplement: Supplementary file 1 [file cancers-13-05813-s001.zip › Supplementary Material (S1).pdf]

WOMEN'S ATTITUDES TO PERSONALISED RISK-BASED SCREENING IN THE NHS BREAST SCREENING PROGRAMME

Summary of Interview Topic Guide

- **Introduction**

PART 1 - CURRENT SCREENING ATTITUDES AND BEHAVIOUR *(c.10 mins)*

1. **Breast screening behaviour/experiences**
- Awareness of NHSBSP?
- Screening behaviour: Routine, occasional, none – why? Pre-eligible - anticipated behaviour
- Feelings about breast screening: Positive/Negative?
- Awareness of and feeling towards benefits and harms of breast screening?

2. **Knowledge of breast cancer, cancer fear & perceived risk**
- Awareness of risk factors for breast cancer?
- Breast cancer worry: Frequency and intensity
- Comparative perceived risk?
- Emotional responses to breast cancer risk: Positive/negative?

PART II – RISK ASSESSMENT *(c.10 mins)*

3. **Measures of risk**

   Clinical measures: Attitudes towards and willingness to be tested for:
- Genetic risk of breast cancer
- Breast Density

   Self-reported: Attitudes towards and willingness to provide information about:
- Family history of breast cancer
- Reproductive history
- Lifestyle behaviours

4. **Cognitive and affective responses for multifactorial risk assessment**
- Understanding and attitudes towards combining individual risk factors?
- Trust in and reliability of individual risk factors: Why?
- Thoughts and feelings about knowing personal level of risk for breast cancer: Positive/negative responses? What and why?

PRESENTATION *(c. 5 mins)*

PART III – RISK-BASED SCREENING *(c.35 mins)*

5. **Age of first and final screening invitation**

   Anticipated cognitive and affective attitudes, practical/other concerns?

6. **Screening Frequency**

   Anticipated cognitive and affective attitudes, practical/other concerns?

7. **Number of risk groups and risk management options**

   Anticipated cognitive and affective attitudes, practical/other concerns?

8. **Final Impressions:**

   Feedback regarding how 3 components risk-stratified screening may look like in practice: risk assessment; risk-stratified screening and preventative treatment options.

Closing the interview

- Thanks for time
- Interview experience
- Additional thoughts or concerns.
- Further information and help-line numbers
- Confidentiality
- What happens next?

INTERVIEW TOPIC GUIDE

- **Introduction to self:** Post-graduate researcher in behavioural science. No medical background or direct involvement with the NHSBSP.
- This breast screening programme is now more than 30 years old, and my research involves looking at some possible ways to improve the service and make it more effective.
- The aim of this interview is to discuss your initial responses to the idea of individually assessing women's risk for breast cancer and explore any thoughts you may have about possible risk-based breast screening options.
- The interview will last for about an hour, and for some of this I will be sharing my computer screen with you. OK? Can I just remind you, that there are no right/wrong answers, we are just throwing ideas around, (i.e. nothing is set in stone). I don't want you to feel that you have to talk about anything that makes you feel uneasy or uncomfortable.
- This study is funded by a breast cancer charity called Breast Cancer Now, which has no direct links with the NHS Breast Screening Programme
- Thank you for signing the Consent Form, thereby agreeing to have this interview recorded. Please be assured that any personal information you provided to us through SAROS will be anonymised, and any details which you inadvertently give us from which it may be possible for someone to identify you, e.g. your postcode or name of your GP will be erased from the interview transcript.
- Please can I remind you that you have the right to withdraw at any point of the interview.
- Before I start the recording, have you got any questions?

PART I – **CURRENT SCREENING ATTITUDES AND BEHAVIOUR**

1.BREAST SCREENING HISTORY, AWARENESS & ATTITUDES TOWARDS NHS Breast Screening Programme (NHSBSP)

(1.i) **Are you aware of the NHSBSP, and please can me a little about what you know about this?** *Probe awareness of who is invited, how often, and why?*

(1.ii) **[IGNORE if pre-eligible] Can you tell me a bit about your experience of breast screening?** *Do you have regular/occasional screens? Don't attend/ pre-eligible? Probe cognitive, emotional, and practical motivations for screening behaviour. What motivates you(not) to accept screening invitation? Do you think you would attend breast screening when eligible?*

(1.iii) **How do you feel about the idea of breast screening?**
Probe whether evokes positive/negative feelings towards the BSP?

(1.iv) **Would you say that you are aware of the benefits of regular breast screening?** *Probe perceived effectiveness of BPS; Prompt: think it prevents women dying from BC?*

(1.v) **Likewise, are you aware of any harms associated with regular breast screening?** *Probe how/where became aware of harms? Influence on motivation to accept screening invitation.*

(1.vi) [IGNORE if not aware of harms] **Given that the benefits of breast screening are thought to outweigh the harms, can you tell me what you feel about these harms?** *Probe how feelings around harms influence screening behaviours and attitudes towards BSP and reasons why?*

2. BREAST CANCER AWARENESS, CANCER WORRY & PERCEIVED RISK:

(2.i) **What do you know or have heard about things that could put some women at a higher risk of breast cancer?** *Probe whether awareness of risk factors influences motivation to attend screening?*

(2.ii) **Are you aware of things you could do, or are already doing, to reduce your risk of breast cancer?** *Probe proactive lifestyle behaviours, e.g., exercise, diet and breast self-examinations.*

BC WORRY

(2.iii) **For some women, breast cancer is something they worry about, while for others it is not. Is this something that you think about much?** *Probe how much, often, and how this compares to general worry about own health?*

PERCEIVED RISK

(2.iv**) Do you have any thoughts about your risk for developing breast cancer compared to other women in your age group?** *Probe why think this? Has this been discussed with GP/other healthcare professional? Think this influences their attitudes and motivation to accept screening invitation?*

(2.v) **What do you feel about your risk of developing cancer?** ***For example, how did me asking the previous question(s) make you feel? Or how does talking about this right now make you feel?***

PART II **– RISK ASSESSMENT**

3. Aims to investigate women's anticipated cognitive and affective attitudes towards each risk factor that would be measured to calculate individual level of risk for breast cancer.

CLINICAL MEASURES

[Genetic tests]: ***Firstly, scientists have now identified some of the genes associated with breast cancer which can be combined into a single genetic risk estimate. This will involve you having a blood or saliva test.***

(3.i) **What are your initial thoughts about having a test to find out your genetic risk for breast cancer?** *Probe whether they would be prepared to have this test? To what extent this would be easy to agree to/ anticipated problems/considerations in decision-making?).*

[Breast Density] *Explain that this is the ratio of breast tissue to fat and that scientists believe dense breast tissue to be a risk factor and that clinicians may use past mammographic images/or measure this at first mammographic screening to determine your breast density]*

(3.ii) **What do you think about allowing clinicians to use previous mammographic images** (*If eligible)*/**your first mammographic scan** *(If pre-eligible)* **to determine the density of your breasts?** *Probe thoughts indicating that this would be difficult/objectionable in any way.*

SELF-REPORTED MEASURES: [*Explain that 3 risk factors could be measured using information provided the interviewee completing questionnaires or filling out some forms, and that we would welcome participant's initial responses to these].*

Family History: [***Explain that risk of breast cancer may be higher if you have multiple first-degree family members who have/had breast cancer, i.e., sister/mother)].***

(3.iii) **Do you think you would be willing to provide details of your family history of breast cancer to help clinicians determine your level of risk?** Probe reasons for positive & negative response.

Reproductive History: [***This refers to age at which women started their periods, age at first pregnancies, number of pregnancies and age of menopause].***

(3.iv) **Again, what do you think about giving clinicians this sort of information to help determine you level of risk for breast cancer?** *Probe reasons for positive & negative response.*

Lifestyle/Health Behaviours

(3.v) **Finally, have you got any thoughts around giving clinicians lifestyle information, such as amount of physical exercise, diet, and alcohol intake?** *Probe reasons for positive & negative response. Are some of these factors easier to consider than others?*

All factors

(3.vi) **Finally, from all these risks factors, so your genetic make-up, breast density reproductive and family history, and lifestyle, are there some that you think are more important, or more reliable, than others?** *Probe reasons for response?*

4. WILLINGNESS TO UNDERGO MULTI-FACTORIAL BREAST CANCER RISK ASSESSMENT Aims: explore willingness to undertake these assessment and attitudes to knowing their own risk level.

Researcher to thank them for the above information and explain that **scientists are now able to input all this information into a computer programme to calculate whether you are at high, average or low risk of breast cancer. This is known as a personalised risk score.**

Check before moving on whether they have any questions or would like any of the above to be explained.

(4.i) **What do you think about the idea of combining your results for all these risk factors to calculate personalised risk level for breast cancer?**

Probe whether this makes sense? Explore reasons why/why not?

(4.ii) **How do you think you would feel about learning your personal level of risk of developing breast cancer?** *Probe why **willing/not willing** to know and any concerns about this? What do they think are the implications of knowing individual level of risk?*

SHARE SCREEN WITH INTERVIEWEE

Slide 1

IDEAL Breast Screening Programme and the NHS BSP currently in practice

It can be useful to envisage the purpose of screening as a form of *sieve* or *mesh.* You can see **all** women between 50 and 70 years are passed through the same screen or mesh every 3 years, i.e., they are not differentiated in any way. You will note that the majority of women pass through the mesh as no abnormalities are detected. These are known as Negative or Normal results, and the benefit here is a reassurance of breast health. A minority of women are 'caught' by the mesh as the screening process has detected abnormalities known as positive results. They are recalled for further tests and receive a diagnosis of breast cancer which is successfully treated. The benefit here is early detection and treatment to save women's lives from breast cancer. So far, we have looked at how the breast screening programme would operate in an ideal world, i.e., there are only benefits and no harms. Unfortunately, in the real world all health screening tests are characterised by a balance between benefits and harms.

ANIMATION [Harms]: First, following further investigation no cancer is detected in around 3 out of 4 positive results. these are known as **False Positive Results.** The harm here is the psychological distress of being recalled for further tests and waiting for the results of these. The second harm is the risk of **overdiagnosis** which is when women receive treatment for a breast cancer than would never have presented a threat to life. In other words, some women will be offered treatment that they may not need. Overall, it is thought that for every 1 woman who has her life saved from breast cancer, about 3 women may be over-diagnosed. Finally, there is a very small risk that **breast screening can miss cancer**. This is rare and may happen in about 1 in 2,500 women screened.

Although most women benefit from the breast screening programme, the potential risk of harm is a cause for concern. This is especially so if you imagine scaling this to population level whereby nearly half a million women aged between 50 and 70 undergo breast screening every year in the UK. Fortunately, breast cancer specialists now believe that the current balance of benefits to harms outlined in this example can be improved. This is where risk-based screening comes into play as it aims to minimize such harms whilst maintaining the benefits of early detection and treatment.

Before, I begin to illustrate how this would work, can I ask you whether you have any questions regarding this so far.

Slide 2:

ANIMATION Risk-Assessment: The risk assessment process which we talked about earlier will allow us to allocate women to risk groups for breast cancer. You can see that most women are identified as average risk [green] and about 20% at low risk [blue]. An estimated 4% will be defined at high risk [brown]. If we were to pass these women through the current breast screening programme mesh, i.e. every 3 years aged between 50 and 70 we would still have the same levels of benefits and harms outlined in the previous slide. Therefore, it

makes sense to think about tailoring the screening mesh to each risk group. I am now going to outline a few of the ways we are thinking about doing this, and it would be great to know what you think and feel about these ideas.

End of presentation.

[Slide 2 cont'd]

ANIMATION -age of screening]: Please bear in mind that any changes are guided by the idea of reducing the harms of screening whilst maintaining its benefits and allocating screening resources to women who are most likely to benefit. So, where there may be suggestions of reducing frequency of screening the aim here is to minimize the harms we have talked about. By contrast, where more frequent screening is proposed the goal is to maximise the ability to target early signs of breast cancer.

5 AGE OF FIRST AND FINAL INVITATIONS FOR BREAST SCREENING: *Researcher to animate/highlight the relevant sections of the **Slide 1** (**Appendix 2 – Interview Slides):** when asking question*

You may have noticed that in the earlier slide outlining the current standard approach that ALL women receive an invitation for breast screening on the basis of their age, so between the age of 50 and 70 (i.e., around 7 invitations for screening over a lifetime). However, this may be too much for women at low risk, or too little for high risk women, it may be an idea to vary the age range between which women receive their first and final invitations for breast screening. Thereby, women with a high risk for breast cancer could start screening earlier (e.g., 40) and end later (e.g., 75). Likewise, low risk women would have their first breast screening later (e.g., 55) and their final screening earlier (e.g., 65). What are your initial thoughts and feelings about this idea?

Probe whether this makes sense? Seem reasonable? Why reasonable or otherwise? Raise any emotional and practical concerns? Do some scenarios appear more manageable than others? Ask them to imagine being identified as average risk and probe what they think and feel about continuing to be screened between 50-70, do they think it should be earlier &/later than this?

6 SCREENING FREQUENCY: *Researcher to animate/highlight the relevant sections of the **Slide 1** (**Appendix 2 – Interview Slides):** when asking question*

ANIMATION – screening frequency: **In addition to varying the age of first and final invitations for breast screening, we could also vary the periods between screening appointments according to individual level of risk. So low risk women would have longer periods between screening. For instance, women at low risk could be screened every 5 years instead of women, and women at high risk could be screened every 12-18 months. Does this make sense to you, and what do you think and feel about this idea?**

Probe whether seems reasonable and why reasonable or otherwise? Raise any emotional and practical concerns? Potential burden of frequent screening? Again, probe what they think about average risk think about continuing to be screened on 3-year basis?

Slide 3

*So far, we have only discussed risk-based screening with 3 risk groups: high, average and low. A further idea would be to increase the number of risk groups to enhance the ability of the screening test to be more precisely targeted to your individual risk score. [ANIMATION – Risk assessment]. Therefore, we have included a **Moderate** risk group to capture individuals at the higher end of the average risk group. You will also note that those at the lowest level of risk are now allocated **Very Low Risk** group. The reasoning for this is that by allocating women to more targeted risk groups, we can reduce the levels of harms from false positive results and overdiagnosis and target signs of breast cancer earlier and with greater accuracy.*

7 NUMBER OF RISK GROUPS & OTHER RISK MANAGEMENT OPTIONS*:*

ANIMATION – 6-12 months: **Please can you try and imagine that you've been identified as <u>high-risk</u> and recommended to have screening every 6 months to a year over a longer period of your lifetime. What are your initial thought and feelings about this?** *Probe whether this makes sense and seems manageable? Do they perceive the benefits of this in terms of higher chance of early detection? Explore emotional responses to this? Would they feel reassured or fearful by more intense levels of surveillance?*

ANIMATION – Lifestyle & risk reducing medication: *Finally, breast screening is only one way of managing your individual risk for breast cancer. There is now evidence that risk-reducing medication (e.g., Tamoxifen) can help to reduce the risk of developing breast cancer. In addition, maintaining a healthy lifestyle, notably reducing alcohol intake, ensuring that your BMI is within healthy limits and taking more physical exercise can reduce your individual level of risk for breast cancer.* **In addition to a more intense screening programme, women identified as <u>high-risk</u> may be advised to take risk-reducing medication and make some changes to their lifestyle. How does this seem to you?** *Probe positive and negative responses to this, e.g., does this level of surveillance seem manageable? What sort of lifestyle changes do they feel would be possible and why? What are their attitudes to risk-reducing medication? Health and emotional concerns?*

ANIMATION – 12-18 months: **If you were identified as <u>moderate-risk,</u> how do you think you would feel about having your breasts screened every year to 18 months over a longer period of your lifetime?** *Probe whether this makes sense and seems manageable? Do they perceive the benefits of this in terms of higher chance of early detection? Explore emotional responses to this? Would they feel reassured or fearful by more intense levels of surveillance given that there is one risk group above this?*

ANIMATION – Lifestyle and consideration of risk reducing medication: **Women in this <u>moderate risk group</u> may be advised to consider taking risk-reducing medication and make some lifestyle changes if necessary. How do you imagine you would think and feel about this?** *Probe positive and negative responses to this, e.g., does this level of surveillance seem manageable? What sort of lifestyle changes do they feel would be possible and why? What are their attitudes to risk-reducing medication? Health and emotional concerns?*

ANIMATION – 3- 4 years: **Bearing in mind that there are now 2 risk groups above you, how do you think you would feel if identified as <u>average risk</u> and invited for screening pretty much the same as you do now, perhaps, every 3-4 years, but over a shorter period of time?** *Probe whether there would still be concerns around*

benefits and harms? Explore whether the fact that there are now 2 risk levels above them make them feel more reassurance? Feel that risk-assessment process should be accompanied by changed screening schedule?

ANIMATION – Lifestyle: **How do you think you would feel about making some changes to your lifestyle to help further reduce your risk-level for breast cancer?** *Probe whether an awareness of risk-management other than screening, influences their attitudes to the prospect of less screening? Explore perceived barriers to lifestyle change. Do they understand why this may help, and feel that this is manageable?*

ANIMATION – No screening + lifestyle: **Finally, please can you imagine that you have a <u>very low risk</u> (note the 'very low' is because there are now 3 risk groups above you). Given what you know about the harms associated with breast screening, how do you think you would respond to the suggestion not to have any screening and instead make any necessary lifestyle changes to maintain this low level of risk?** *Probe whether this makes sense? Accepted as a reasonable option? Explore health/emotional concerns raised by this option, e.g., apprehension versus relief a prospect of no screening, appreciation of minimisation of harms? Barriers to lifestyle change?*

8. **FINAL IMPRESSIONS:**

You can now see how during this interview; we have put together an example of how risk-based screening could look in practice. We have discussed its 3 components: risk assessment risk-based screening strategies and risk reducing options, and it would be great to know if you have any final comments or concerns about any of these aspects? *Probe which aspects/features make the most intuitive sense? Perceived effectiveness in terms of balance of benefits-harms? Levels of intervention coherence and perceived emotional, cognitive and logistical barriers?*

CLOSE THE INTERVIEW

- Thank you and how have you found the interview? [Researcher to check that no areas of the interview caused undue distress, and to clarify any areas of concern & reiterate the hypothetical nature of the suggested screening scenarios]
- Additional thoughts or concerns?
- Further information and help-line numbers (BCN – On-line forum:
- Reassure about confidentiality and repeat info provided at the beginning
- Give details of how £50 voucher will be provided?
- Offer to keep them informed regarding study results.
